# Functionalization of Screen-Printed Electrodes with Grape Stalk Waste Extract-Assisted Synthesized Silver and Gold Nanoparticles: Perspectives of Electrocatalytically Enhanced Determination of Uranyl Ion and Other Heavy Metals Ions

**DOI:** 10.3390/nano13061055

**Published:** 2023-03-15

**Authors:** Karina Torres-Rivero, Antonio Florido, Vicenç Martí, Julio Bastos-Arrieta

**Affiliations:** 1 Departament d’Enginyeria Química, Escola d’Enginyeria de Barcelona Est (EEBE), Universitat Politècnica de Catalunya, BarcelontaTEch (UPC), Av. Eduard Maristany 16, 08019 Barcelona, Spain; 2 Barcelona Research Center for Multiscale Science and Engineering, Av. Eduard Maristany 16, 08019 Barcelona, Spain; 3 Departament d’Enginyeria Química i Química Analítica, Facultat de Química, Universitat de Barcelona, Martí i Franquès 1-11, 08028 Barcelona, Spain; 4 Institut de Recerca de l’Aigua (IdRA), Universitat de Barcelona (UB), 08028 Barcelona, Spain

**Keywords:** screen-printed electrodes, nanoparticles, green synthesis, sensor, differential pulse voltammetry, uranyl detection, Pb detection, Cd detection

## Abstract

Recently, nanotechnology and nanoparticles (NPs) such as AgNPs and AuNPs have become important in analytical chemistry due to their great potential to improve the performance of electrochemical sensors. In this work, Ag and Au nanoparticles have been synthesized using a green route in which a grape stalk waste extract is used as a reducing agent to obtain metallic nanoparticles. These NPs were used to customize the surface of commercial screen-printed electrodes (SPCNFEs). The spin-coating method was used to modify commercial SPCNFEs under a nitrogen atmosphere. The resulting electrodes were used in a determination study of Cd(II), Pb(II), and U(VI) with differential pulse anodic stripping voltammetry (DPASV). The customized green AgNPs and AuNPs electrodes presented higher sensitivity and electroanalytical performance than the non-modified SPCNFE. The results showed that the best analytical parameters were obtained with the green, silver nanoparticle SPCNFEs, with a LOD of 0.12 μg L^−1^ for Pb(II), which is a lower value compared to the most restrictive regulation guidelines. Additionally, the U(VI) ion was successfully determined using the developed G-AgNPs-SPCNFE in spiked tap water, showing comparable results with the ICP-MS technique.

## 1. Introduction

Recently, significant efforts have been made to synthesize metallic nanoparticles using environmentally friendly methodologies. Many investigations have proven the critical role of microorganisms and biological systems in producing metal nanoparticles [1,2,3,4,5].

The green synthesis of metallic nanoparticles can involve different organisms such as algae, fungi, bacteria, diatoms, or plants. They possess phytochemicals, including proteins, metabolites, sugars, flavonoids, or polyphenols, and can transform metal ions into metal nanoparticles due to their reducing capability [6,7,8,9,10].

These “green” nanoparticles obtained from the combination of biological reducing agents and metal salt precursors can develop the electrochemical sensors by improving the analytical response and determining specific analytes, particularly heavy metal ions (HMIs).

There exist well-established analytical techniques (inductively coupled plasma mass spectroscopy (ICP-MS), inductively coupled plasma optical emission spectroscopy (ICP-OES), etc.) to determine the concentration of HMI in different media [9]. They have the advantage of having high sensitivity towards HMI and high selectivity. However, they require costly equipment and specialized technicians, increasing the analytical costs. These factors have augmented the interest in alternative analytical techniques, such as electrochemical methods, which are more advantageous for in situ measurements and have lower analytical costs. Therefore, electrochemical techniques, specifically anodic stripping voltammetry (ASV), have been recognized as better alternatives for determining trace HMI because they do not require costly equipment, specialized personnel, and high costs [9,10]. These methods present a high sensitivity and detection capacity [9,11,12].

Some investigations have studied the voltammetric determination of Cd(II) [13,14,15] and Pb(II) [16,17,18], the most common target analytes, using modified screen-printed electrodes (SPEs) with biosynthesized nanoparticles. Although this study is also focused on the voltammetric determination of lead and cadmium, another analyte is incorporated due to its significance in nuclear industry safety, U(VI). Some investigations have reported the use of glassy carbon electrodes modified with chemically synthesized gold nanoparticles obtaining a limit of detection of 0.3 µg L^−1^ [19], and a nanocomposite carbon paste electrode functionalized with gold nanoparticles allowed for an LOD of 0.49 µg L^−1^ by cyclic voltammetry [20]. However, no investigations have reported the use of biosynthesized nanoparticles to modify electrochemical sensors to determine U(VI) in aqueous samples.

On the other hand, inductively coupled plasma-atomic emission spectrometry, ICP-AES, and fluorometry, among other techniques, are used to determine uranium as major, minor, and trace elements in samples. The limits of detection achieved with these highly specialized analytical techniques are between 5 and 20 ng L^−1^ [21,22].

As the previous works reported, the modified electrodes facilitate the detection and quantification of the specified HMI under aqueous conditions. However, the working concentration ranges of the obtained electrodes are higher than the regulated concentrations of Cd(II) and Pb(II) for drinking water, according to the World Health Organization. These values are 3 µg L^−1^ for cadmium and 10 µg L^−1^ for lead [23], and the from European Union Directive 2020/2184, which are 5 µg L^−1^, 10 µg L^−1^, and 30 µg L^−1^ for cadmium, lead, and uranium, respectively [24].

Therefore, this work proposes the modification of screen-printed carbon nanofiber electrodes (SPCNFEs) with green synthesized silver and gold nanoparticles using the extract of grape stalk wastes. The resulting electrodes will be electrochemically characterized to detect and quantify U(VI), Pb(II), and Cd(II) in aqueous media at a trace level. 

## 2. Materials and Methods

### 2.1. Reagents and Materials

Grape stalk waste from wine production was supplied by a winery cooperative (Subirats, Alt Penedès, Spain). The grape stalk extract was prepared and optimized to perform the nanoparticle synthesis, as reported by [25].

Silver nitrate for the silver nanoparticle preparation was purchased from Merck (Darmstadt, Germany), while tetrachloroauric(III) acid trihydrate for the gold nanoparticle synthesis was obtained from Acros Organics (Barcelona, Spain). U(VI) (1 mg L^−1^) was prepared using a uranyl nitrate ICP standard. Pb(II) (1 mg L^−1^) and Cd(II) (1 mg L^−1^) solutions were prepared by performing the appropriate dilution of ICP standards solutions purchased from Panreac Applichem (Castellar del Vallès, Spain), and they were standardized by ICP-OES. A 0.1 mol L^−1^ acetic acid/acetate buffer (pH 4.5) was prepared from acetic acid (Merck, Munich, Germany) and sodium acetate (Panreac, Barcelona, Spain) and used as an electrolyte for a constant pH and to avoid the formation of metal hydroxo complexes.

Potassium hexacyanoferrate (II) trihydrate (K_4_Fe(CN)_6_·3H_2_O), potassium hexacyanoferrate (III) (K_3_Fe(CN)_6_ were purchased from Merck (Darmstadt, Germany) to perform the electrochemical impedance spectroscopy studies. Ultrapure water (Milli-Q plus system, Millipore, Billerica, MA, USA) was used to prepare all of the solutions and suspensions.

Tap water samples were collected from the local water distribution network managed by Aigües de Barcelona Company (Barcelona, Spain; https://www.aiguesdebarcelona.cat/, accessed on 1 February 2023), mainly using potable water obtained from the treatment of Llobregat and Ter Rivers.

All the glassware and magnetic stir bars were thoroughly cleaned using aqua regia (HCl/HNO_3_ 3:1, *v*/*v*) and rinsed with Milli-Q water.

### 2.2. Apparatus

A multi Autolab/M204) potentiostat/galvanostat (Metrohm, Herisau, Switzerland) was used to perform the electrochemical characterization. For data acquisition, NOVA 2.1.2 software was used. Pt wire and Ag/AgCl/ KCl 3 mol L^−1^ (Metrohm, Herisau, Switzerland) were used as auxiliary and reference electrodes, respectively. The working electrode was a carbon nanofiber-modified screen-printed electrode of 4 mm diameter (SPCNFE) from Metrohm (Herisau, Switzerland), modified with green silver and gold nanoparticles and connected to the potentiostat with a cable (ref. CAC, Dropsens). 

A Crison GLP-22 pH-meter (Alella, Barcelona, Spain) was used for the pH measurements.

To analyze the surface plasmon resonance (SPR) of the biosynthesized Ag-NPs and Au-NPs, UV-Vis spectrums of the colloidal suspensions were recorded using a spectrometer Flame S-UV-VIS-ES from Ocean Insight (Orlando, FL, USA). 

The nano tracking analysis measurements (Brownian motion analysis) for particle size determination were performed using a NanoSight NS3000 at 25 °C at a laser wavelength of 488 nm. For Brownian motion analysis, the samples were diluted. Between 2000 and 2200 tracks were evaluated for each sample.

The prepared standards solutions were standardized using the ICP-OES model 5100 or by ICP-MS model 7800 from Agilent Technologies (Santa Clara, CA, USA).

### 2.3. Silver and Gold Nanoparticles Synthesis Using Grape Stalk Extract as a Reducing Agent

After extract preparation, the green synthesis of AgNPs (G-AgNPs) and (G-AuNPs) was performed, following the procedure proposed in [26,27].

Solutions of AgNO_3_ and HAuCl_4_ 0.01 M were used as the NPs precursor. A volume of 6 mL of the previous solutions was added to 8 mL of the freshly prepared extract. The test tubes were covered and stirred to obtain a homogeneous solution. To eliminate nanoparticles that were larger than 100 nm, the tubes were centrifuged at 4000 rpm for 20 min. Finally, the supernatant was removed from the tubes and filtered using a syringe filter to eliminate any precipitate. The resulting solution was refrigerated at 4 °C Z-potential of the NPs and measured using a Zetasizer Nano Z from Malvern Instruments Inc., (Malvern, UK).

### 2.4. Screen-Printed Electrode Modification

The SPCNFE modification using the synthesized NPs was performed by employing a WS-650-8B spin-coater from Laurell Technologies Corporation (North Wales, PA, USA).

The surface of the screen-printed carbon nanofiber electrode (SPCNFE) was modified using the procedure explained in [28]. A volume of 20 μL of the nanoparticle suspension was placed onto the sensor surface and then placed into the spin coater equipment. A cycle of 3000 rpm for 3 min under a nitrogen atmosphere and a vacuum was used. This procedure was repeated twice to modify the electrode surface properly.

### 2.5. Screen-Printed Electrode Surface Characterization

G-AgNPs and G-AuNPs, as well as the surface morphology of the SPCNFE electrodes, were characterized using a JEM-1400 transmission electron microscope (TEM) from JEOL (Tokyo, Japan) and a Gemini scanning electron microscope (SEM) from ZEISS^®^ (Jena, Germany). TEM and STEM images were used to determine the size distribution of the obtained G-AgNPs and G-AuNPs. The size distribution histograms were calculated using Image-J version 1.51m software (National Institutes of Health (NIH, Bethesda, MD, USA).

### 2.6. Green-AgNPs and Green-AuNPs Modified SPCNFE Electrochemical Characterization

#### 2.6.1. Cyclic Voltammetry Measurements

The electrochemical performance of the G-AgNPs- and G-AuNPs-modified electrodes was tested using the cyclic voltammetry (CV) technique. The electrochemical properties of the bare SPCNFE were compared to the electrochemical properties of the modified electrodes. The cyclic voltammograms were recorded in 5 mM of [Fe(CN)_6_]^−3/−4^ redox pair prepared in 0.1 mol L^−1^ acetic acid/acetate buffer pH 4.5. The scanned potential was from −0.6 V to 0.6 V, with a scan rate of 0.05 V/s.

#### 2.6.2. Electrochemical Impedance Spectroscopy (EIS) Studies

The Nyquist diagrams were recorded in a solution containing 5 mM [Fe(CN)_6_]^−3/−4^ and 0.1 mol L^−1^ KCl. The studied frequency ranged from 10 Hz to 1000 kHz with an alternating current (AC) amplitude of 10 mV.

#### 2.6.3. Differential Pulse Anodic Stripping Voltammetry (DPASV) Measurements to Determine Heavy Metals

The G-AgNPs- and G-AuNPs-modified electrode behavior was tested to determine Cd(II), Pb(II), and U(VI) in aqueous samples.

All the metal ions’ measurements were performed with differential pulse anodic stripping voltammetry with a deposition time of 300 s at a −1.2 V deposition potential. Voltammograms were obtained by scanning the potential between −1.2 V and −0.2 V, using a step potential of 5 mV, pulse amplitudes of 50 mV, and a pulse time of 50 ms.

All the metal ions’ measurements were performed with differential pulse anodic stripping voltammetry with a deposition time of 300 s at a −1.2 V deposition potential. Voltammograms were obtained by scanning the potential between −1.2 V and −0.2 V, using a step potential of 5 mV, pulse amplitudes of 50 mV, and a pulse time of 50 ms.

#### 2.6.4. Determination of Heavy Metals Ions in Real Water Samples

The tap water samples were spiked with 60 µg L^−1^ of U(VI). To perform the voltammetric determination of the metal ions, te samples were acidified with 0.1 mol L^−1^ acetic acid/acetate buffer pH 4.5, resulting in a final concentration of 30 µg L^−1^ for U(VI). Three additions were made from a standard solution of 1 mg L^−1^ of U(VI). DPASV measurements were recorded using the experimental conditions mentioned in the previous section. 

## 3. Results

### 3.1. Grape Stalk Waste Extract Assisted-Synthesis Green-AgNPs and Green-AuNPs Characterization

#### 3.1.1. Spectrophotometric Characterization by UV-Vis

**Figure 1:**
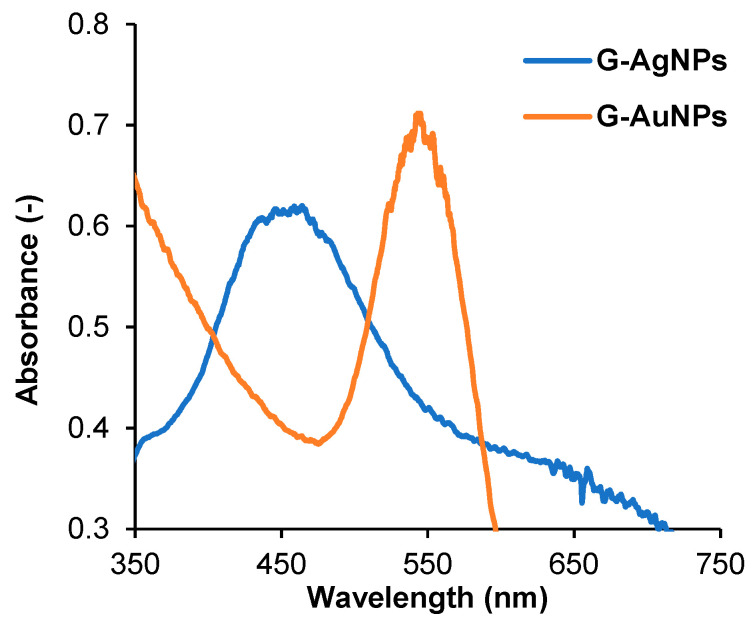
UV-Vis spectra of green synthesized silver nanoparticles (G-AgNPs) (blue line) and gold nanoparticles (G-AuNPs) (orange line).

Figure 1 shows the UV-Vis spectra of silver and gold synthesized nanoparticles due to the possibility of perceiving these nanoparticles’ formation by simple observation of the colloidal suspension color. For silver nanoparticles (G-AgNPs), the absorbance peak is shown at 450 nm, while for the gold nanoparticles (G-AuNPs), the absorbance peak shifted to a higher wavelength value, 550 nm. Additionally, for G-AgNPs, the absorbance peak is wider than the G-AuNPs absorbance peak due to the silver nanoparticles being more polydisperse, and in turn, generating a stronger interaction with the UV light. 

The wavelength value agreed with the value reported in the bibliography, in which silver colloids exhibited a maximum absorbance of between 400 and 500 nm due to the surface plasmon resonance phenomena [29,30]. Regarding the G-AuNPs, the gold nanoparticle colloids exhibited a maximum absorbance between 514 and 550 nm [31,32], which is in concordance with the wavelength values reported by this investigation.

#### 3.1.2. Morphological Characterization by Means of Scanning and Transmission Electron Microscopy

**Figure 2:**
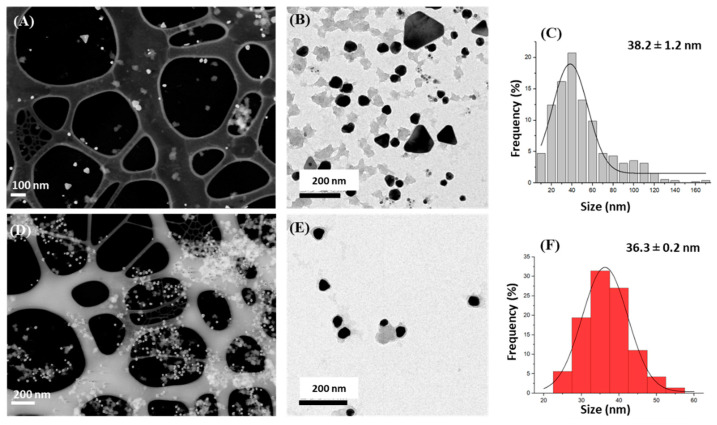
Scanning and Transmission electron microscopy (STEM) micrographs of the (**A**) green silver nanoparticles (G-AgNPs) and (**D**) green gold nanoparticles (G-AuNPs), Transmission electron microscopy of (**B**) G-AgNPs and (**E**) G-AuNPs, and their corresponding size histograms for (**C**) G-AgNPs and (**F**) G-AuNPs.

The synthesized green silver (Figure 2A,B) and green gold (Figure 2D,E) nanoparticles were characterized using scanning and transmission electron microscopy (STEM and TEM), respectively, to determine the size and shape of the obtained NPs.

The adequacy of the grape stalk extract for the synthesis of AgNPs and AuNPs was confirmed through the direct observation of the STEM and TEM micrographs.

From the TEM micrographs (Figure 2B,E), a population of 500 nanoparticles was measured to obtain a size distribution histogram of the synthesized silver and gold nanoparticles (Figure 2C,F). NPs counting and data treatment were performed as reported previously [16]. 

The results reveal that G-AgNPs and G-AuNPs exhibited a mean size of (51.4 ± 2.3) nm and (36.3 ± 0.2) nm, respectively. For the silver nanoparticles, round and prism particles were observed, while the gold nanoparticles exhibited mainly a round shape.

In addition, the Z potential value was measured for both nanoparticle suspensions to investigate their stability; values of −23.93 ± 5.41 mV and −29.29 ± 6.80 mV were obtained for G-AgNPs, and G-AuNPs, respectively.

#### 3.1.3. Nano Tracking Analysis of the Synthesized G-AgNPs and G-AuNPs

The average size and concentration of the synthesized AgNPs and AuNPs were also estimated using nano tracking analysis (NTA). The results indicate size values of 115 ± 13 nm and 135 ± 6 nm, and concentrations of 4.83 × 10^10^ and 9.76 × 10^8^ particles/mL for G-AgNPs and G-AuNPs, respectively. The differences between the size of G-AgNPs and G-AuNPs by STEM (51 and 36 nm) and NTA are due to both techniques being based on different physical principles. Thus, the STEM technique can help determine the diameter of the NPs’ metallic core, while the NTA technique measures the hydrodynamic diameter in the solution, and, in this case, the obtained nanoparticle size has a larger value, as is reported in the literature [33].

### 3.2. G-AgNPs and G-AuNPs Modified SPNCFE Scanning Electron Microscopy Characterization

Nanoparticles play an essential role in terms of their improved properties in sensing and biosensing technologies [34,35]. Consequently, green Ag-NPs and green Au-NPs were incorporated into commercial screen-printed electrodes due to their capacity to increase the electrocatalytically active zones on the structures of the composite material [16]. 

**Figure 3:**
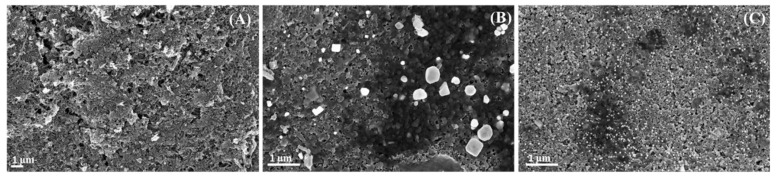
Scanning electron microscopy micrographs of (**A**) bare screen-printed carbon nanofiber electrode (SPCNFE), (**B**) G-AgNPs-SPCNFE, and (**C**) G-AuNPs-SPCNFE.

In Figure 3A, an image of the bare SPCNFE is shown. In addition, SEM images of the modified surface of SPCNFE with green Ag-NPs (Figure 3B) and green Au-NPs (Figure 3C) are shown. Both micrographs of the modified sensors confirmed the actual attachment of the G-AgNPs and G-AuNPs (white dots) onto the electrode carbon nanofibers.

Hence, all of the SPCNFEs were successfully modified with the corresponding nanoparticles. This could explain the capability to improve electrochemical performance compared to the non-modified electrodes, as observed in other electrochemical systems modified with nanomaterials [36].

### 3.3. Modified Screen-Printed Electrode with G-AgNPs and G-AuNPs Electrochemical Performance

#### 3.3.1. Cyclic Voltammetry Studies

**Figure 4:**
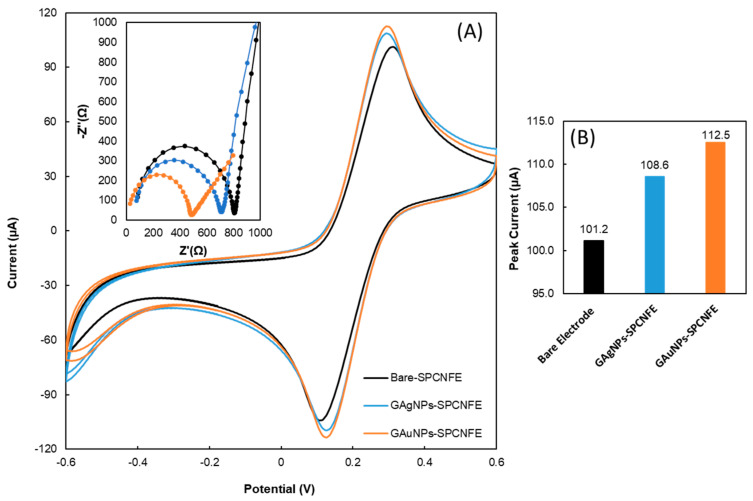
(**A**) Cyclic voltammograms, Inset: Nyquist diagram (recorded in 5 mmol L^−1^ [Fe(CN)_6_]^−3/−4^ and 0.1 mol L^−1^ KCl), and (**B**) bar graph of the bare screen-printed carbon nanofibers electrodes (SPCNFE), green synthesized silver and gold nanoparticles modified SPCNFE in 5 mmol L^−1^ [Fe(CN)_6_]^−3/−4^ in acetic acid/acetate buffer 0.1 mol L^−1^ pH 4.5.

In this study, the green synthesized silver nanoparticles and gold nanoparticles were deposited onto the screen-printed carbon nanofiber electrode surface and were analyzed using cyclic voltammetry with the experimental conditions explained in the previous section. Based on Figure 4, the peak current of the G-AuNPs-modified screen-printed carbon nanofiber electrodes (G-AuNPs-SPCNFEs) increased by 112.5 µA relative to the bare SPCNFE. Additionally, it was 4% higher compared to the G-AgNPs-SPCNFE (108.6 µA). These peak current increments could explain the enhanced electrocatalytic activity and larger surface area of AgNPs and AuNPs, improving the mass transport rate of electrons between the sensor surface and [Fe(CN)_6_]^−3/−4^ ions and consequently inducing faster electron transfer kinetics.

#### 3.3.2. Electrochemical Impedance Spectroscopy Studies and Electroactive Surface Area Determination

The effect of G-AgNPs and G-AuNPs on the screen-printed carbon nanofiber electrodes was studied using electrochemical impedance spectroscopy (EIS). The inset in Figure 4 shows the Nyquist diagrams obtained for the bare SPCNFE and the G-AgNPs- and G-AuNPs-modified SPCNFE fitted to the Randle circuit. In Table 1, it is possible to observe the value of the charge transfer resistance (R_ct_) for all of the studied electrodes, which was obtained as the semicircle diameter of the Nyquist spectra. The R_ct_ decreased by 14% and 28% for the modified SPCNFEs with the G-AgNPs and G-AuNPs, respectively, compared to the R_ct_ value for the non-modified SPCNFE, indicating a higher electrocatalytic response of the nanoparticle modified electrodes. Additionally, the electroactive area of the electrodes was calculated using the Randles–Sevcik equation. As can be observed, the A_T_ increased as the corresponding R_ct_ values decreased, thereby confirming the occurrence of an enhanced reaction rate at the electrode surface.

#### 3.3.3. Testing Analytical Performance towards Pb(II), Cd(II), and U(VI) Voltammetric Determination

**Figure 5:**
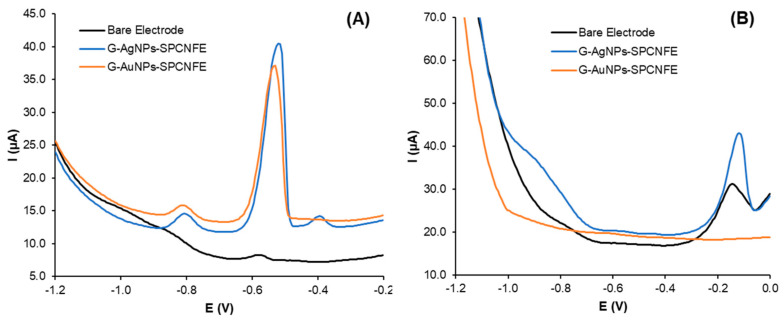
Comparative study using the bare electrode and the green nanoparticles modified electrode for a fixed concentration of 75 µg L^−1^ of (**A**) Cd(II) and Pb(II) analytes and (**B**) U(VI) analyte.

Before the voltammetric determination of Pb(II), Cd(II) (Figure 5A), and U(VI) (Figure 5B) using the G-AgNPs and G-AuNPs modified electrodes, a comparative study between the bare and the modified electrodes with a fixed concentration of 75 µg L^−1^ for each metal ion was performed. The electrode modification with the green-synthesized nanoparticles results in an important signal increase for every heavy metal ion evaluated, which is vital for the determination of heavy metals at low concentration levels.

At this point, an electrochemical characterization using green silver (G-AgNPs) and green gold (G-AuNPs) nanoparticles modified electrodes to determine Pb(II) and Cd(II) was performed. For the U(VI), only the G-AgNPs-SPCNFE was used to implement the voltammetric determination due to the lack of enhancement in terms of response using the G-AuNPs-SPCNFE compared to the non-modified electrode.

- Pb(II) determination

**Figure 6:**
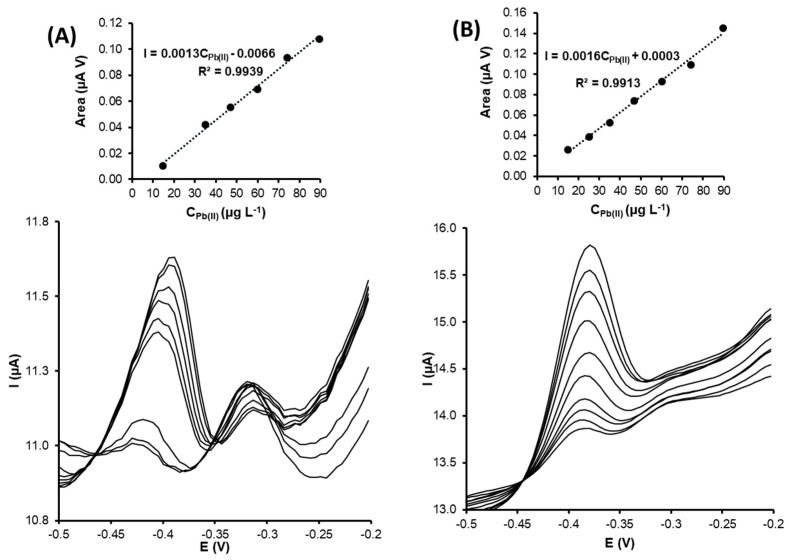
DPASV voltammograms for Pb(II) determination and its calibration curves (top inset) for (**A**) G-AgNPs-SPCNFE and (**B**) G-AuNPs-SPCNFE in acetic acid/acetate buffer 0.1 mol L^−1^ pH 4.5 using an Ed of −1.2 V and a td of 300 s.

Figure 6A,B show the voltammograms and calibration plots obtained for Pb(II) detection using green-AgNPs-based and green-AuNPs-based electrodes. In both graphics, it is possible to relate the peak area to the applied potential between −1.2 V and −0.2 V.

Calibration curves by differential pulse anodic stripping voltammetry (DPASV) were obtained by increasing the Pb(II) concentration to between 1.0 and 90.0 µg L^−1^ and using the experimental conditions explained in the experimental section. The Pb(II)-related peak using G-AgNPs-SPCNFE and G-AuNPs-SPCNFE was observed at around −0.40 V. 

The obtained analytical parameters, such as limits of detection (LOD), the limit of quantification (LOQ), sensitivities, and linear ranges using both modified electrodes (G-AgNPs-SPCNFE and G-AuNPs-SPCNFE), are listed in Table 2. Limits of detection and quantification for differential pulse voltammograms (DPV) calibration curves were calculated using the Miller and Miller methodology [37]. The LOQ was considered to be the lowest limit of the linear range. 

Both modified electrodes (G-AgNPs-SPCNFE and G-AuNPs-SPCNFE) exhibited excellent and similar behavior regarding the voltammetric determination of Pb(II). The obtained LOD for green silver and gold nanoparticles based on screen-printed carbon nanofiber electrodes was 0.12 µg L^−1^ (see Table 2). 

Lower LOD values were obtained compared to other LODs reported in the literature. Amare et al. [38] developed a sensor modified with silver nanoparticles that was synthesized using Ocimum Sanctum (commonly known as basil) leaf extract. The modified electrode was used to perform voltammetric determination of Pb(II) and other heavy metal ions. The detection limit was estimated at 48 µg L^−1^. However, the reported linear range was wider, from 5x10^3^ to 1.6x10^5^ µg L^−1^, compared to the one obtained in this investigation (0.39–40 µg L^−1^). Another investigation developed a gold nanoparticles-modified screen-printed electrode to determine Pb(II) in seawater with an LOD of 0.06 µg L^−1^, showing linearity in a more restricted range (4.1–24.9 µg L^−1^) that the one found in this work [39] (see Table 3). 

The sensitivities, calculated as the slope of the calibration plots, showed very close values for both modified electrodes. The G-AuNPs-SPCNFE sensitivity (1.5) was 15% higher than the sensitivity value for G-AgNPs-SPCNFE (1.3). This can be explained by the fact that a higher concentration of AuNPs was present in the sample, as determined after performing the NTA technique (see Section 3.1.3), which can be interpreted as being due to a higher sensitivity resultant of the enhanced catalytic activity of the modified electrode.

- Cd(II) determination

Cadmium detection was performed by using green silver and gold nanoparticle customized electrodes.

**Figure 7:**
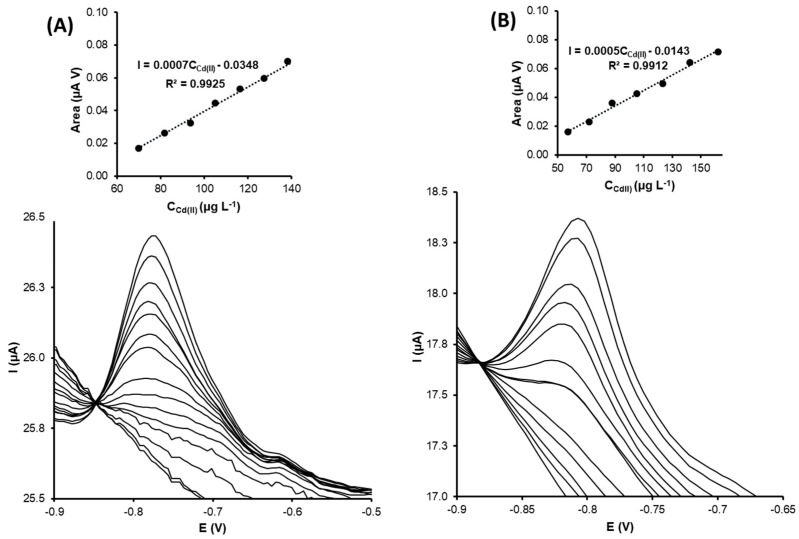
DPASV voltammograms for Cd(II) determination and its calibration curves (top inset) for (**A**) G-AgNPs-SPCNFE and (**B**) G-AuNPs-SPCNFE in acetic acid/acetate buffer 0.1 mol L^−1^ pH 4.5 using an Ed of −1.2 V and a td of 300 s.

Figure 7A,B show the cadmium determination voltammograms using G-AgNPs-modified and G-AuNPs-modified SPCNFEs, respectively. In both cases, the Cd(II) stripping peak was around −0.8 V. The calibration plots confirmed that the peak area increased as the cadmium concentration increased with good correlation coefficient values. 

Calibration curves by differential pulse anodic stripping voltammetry (DPASV) were obtained by increasing the Cd(II) concentration to between 4.0 and 162.0 µg L^−1^. The obtained detection limit for the green silver nanoparticles modified electrode was 12.1 µg L^−1^, while the LOD for the green gold nanoparticles modified electrode was 18.2% higher (14.3 µg L^−1^) (see Table 2). 

Amare et al. [38] reported the biosynthesis of silver nanoparticles using Ocimum sanctum (holy basil) and modified a carbon-pasted electrode to determine Cd(II) in a textile discharge effluent. The detection limit was found to be 89 µg L^−1^, a much higher value than the LOD obtained in this investigation (12.1 µg L^−1^). In addition, the reported linear range (1–80 µg L^−1^) is less restricted regarding the upper concentration limit than the one obtained in this study (40 µg L^−1^). Lu et al. [14] customized screen-printed carbon electrodes with star-shaped gold nanoparticles for the voltammetric determination of Cd(II) and other trace elements. The Cd(II) linear stripping response was over the concentration range of 0 to 100 µg L^−1^, with an obtained limit of detection of 1.6 µg L^−1^ (see Table 3). 

The sensitivities for G-AgNPs-SPCNFE and G-AuNPs-SPCNFE regarding the Cd(II) determination were 0.74 (0.03) and 0.54 (0.02), respectively.

Finally, the reproducibility was verified for both metal ions, and relative standard deviations (RSDs) of 4.4% and 2.9% for Pb(II) and Cd(II) were obtained, respectively.

- Uranyl ion determination

**Figure 8:**
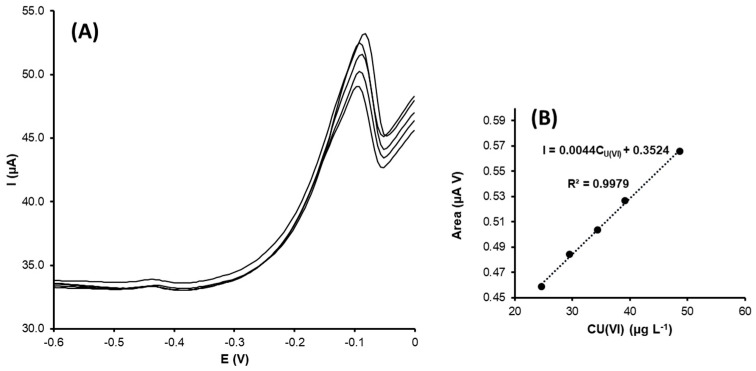
(**A**) DPV voltammograms for U(VI) and (**B**) calibration plot obtained using G-AgNPs-SPCNFE in acetic acid/acetate buffer 0.1 mol L^−1^ pH 4.5 using an E_d_ of −1.2 V and a t_d_ of 300 s.

As previously stated, uranyl ion voltammetric determination was performed using G-AgNPs-SPCNFE. In Figure 8, the DPV calibration curves corresponding to the U(VI) and its calibration plot are shown. 

The stripping peak corresponding to uranyl was observed at around −0.15 V. The calibration curves were recorded using DPASV, and the uranyl ion concentration increased from 5 to 49 µg L^−1^. As in the previous cases, the calibration plot confirmed that the peak area increased with the concentration, showing a good correlation coefficient value. The obtained limit of detection was 5.2 µg L^−1^.

The sensitivity value for the modified electrode regarding the U(VI) determination was around 4.3 (0.2), a higher value compared to the obtained electrode sensitivities for the Cd(II) and Pb(II) determination, but with a more restricted linear range. Additionally, the reproducibility of the developed sensor was estimated at 2.9% RSD. 

No similar methods have been reported in the literature (based on green NPs electrodes) to determine uranyl ions in water. However, some investigations have developed different types of electrochemical sensors. For example, Pinaeva et al. [40] developed a nanostructured membrane to isolate uranyl (VI) from aqueous solutions and performed further determination using cathodic stripping voltammetry with a detection limit of 17 μg L^−1^ in a linear range from 20 to 100 μg L^−1^ (see Table 3). 

Table 2 shows all the analytical parameters obtained from the DPV voltammograms and their corresponding calibration curves. The limits of detection (LOD) were calculated as three times the standard deviation of the blank solution divided by the slope of the calibration curve [37].

**Table 2:** Analytical parameters obtained for G-Ag-NPs and G-Au-NPs modified sensors.

	G-AgNPs-SPCNFE			G-AuNPs-SPCNFE	
	Pb(II)	Cd(II)	U(VI)	Pb(II)	Cd(II)
LOD (µg L^−1^)	0.12	12.1	5.2	0.12	14.3
LOQ (µg L^−1^)	0.39	40.2	17.4	0.40	47.5
Linear Range (µg L^−1^)	0.39–40	40.2–149	17.4–49	0.4–90	47.5–162
Sensitivity (SD) (nA V)	1.3 (0.1)	0.74 (0.03)	4.3 (0.2)	1.5 (0.1)	0.54 (0.02)

(SD): Standard deviation.

In Table 3, a comparison of previous studies using similar types of silver or gold- NPs-modified electrodes is presented with the developed sensors in this investigation. 

**Table 3:** Comparison between this investigation with previous studies using different electrochemical systems to determine the investigated heavy metal ions.

Analyte	Voltammetric Electrochemical Sensor	Detection Limit (µg L^−1^)	Linear Range (µg L^−1^)	Ref.
Pb(II)	AgNPs-Carbon paste electrode	48	5 × 10^3^–1.6 × 10^5^	[38]
	G-AgNPs-SPCNFE	0.12	0.39–40	This study
Cd(II)	AgNPs-Carbon paste electrode	89	5 × 10^3^–1.6 × 10^5^	[38]
	G-AgNPs-SPCNFE	12.1	40.2–149	This study
Pb(II)	AuNPs/polyanilinemulti-walled carbon nanotubes-SPE	0.037	1–180	[41]
	AuNPs-SPCE	0.06	4.1–24.9	[39]
	G-AuNPs-SPCNFE	0.12	0.4–90	This study
Cd(II)	GO/multi-walled carbon nanotubes/AuNPs *	0.7	1–80	[42]
	AuNPs-SPCE	1.6	0–100	[14]
	G-AuNPs-SPCNFE	14.3	47.5–162	This study
U(VI)	PVDF films/track etched PVDF membranes **	17	20–100	[40]
	G-AgNPs-SPCNFE	4.3	17.4–49	This study

* GO: Graphene oxide; ** PVDF: polyvinylidene fluoride films.

The NPs-modified sensors found in the bibliography have been customized using obtained nanomaterials by chemical synthesis and additional materials and or polymers for analytical purposes. Consequently, this study proposes that functionalization using green silver and gold nanoparticle electrodes has numerous advantages compared to previous investigations, which can be enumerated with its simple electrode modification technique, good stable nanoparticles dissolution, and environmentally friendly nanoparticle synthesis method, supposing the reutilization of a residue with the possibility of scaling-up for large-scale fabrication. 

#### 3.3.4. Application of the G-AgNPs-SPCNFE, and G-AuNPs-SPCNFE Modified Electrodes to the Analysis of a Real Sample Containing U(VI)

From the analytical parameters of G-AgNPs-SPCNFE, it can be concluded that this modified electrode exhibited better performance compared to G-AuNPs-SPCNFE; consequently, the silver nanoparticle-modified electrode was chosen to perform the U(VI) determination in spiked tap water. It should be noted that DPASV measurements of non-spiked tap water did not present any uranyl signals. 

Regarding G-AuNPs-SPCNFE, the determination of U(VI) was unsuccessful (Figure S2, Supporting Information (SI)); for this reason, the G-AgNPs-SPCNFE electrode was selected for the further determination of U(VI) in the real sample. 

**Figure 9:**
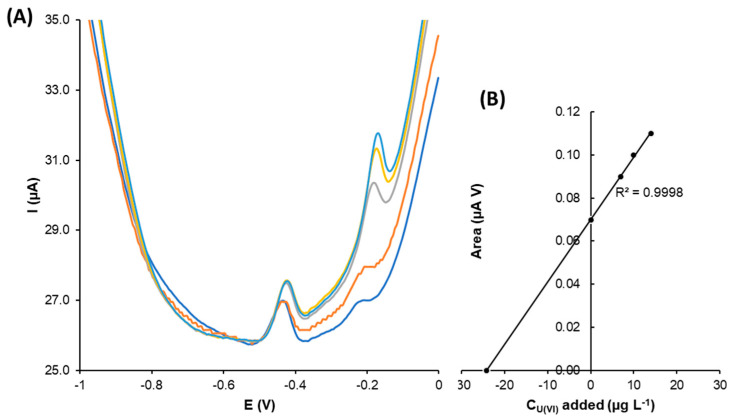
(**A**) DPASV voltammograms obtained for uranyl ion in spiked tap water using G-AgNPs-SPCNFE in acetic acid/acetate buffer 0.1 M pH 4.5 with an E_d_ of −1.2 V and a t_d_ of 300 s, and (**B**) uranyl standard addition curve.

In Figure 9, the obtained DPASV voltammograms for the uranyl spiked tap water samples using the G-AgNPs-SPCNFE are shown. As can be observed, well-shaped U(VI) peaks were achieved as the uranyl concentration increased, which was confirmed by the calibration curve, showing an R^2^ value of 0.9998.

Three replicates were considered for the analysis of U(VI) tap water samples using the green-silver nanoparticles-modified electrode. The result was 26.34 µg L^−1^ (SD: 1.91 µg L^−1^). To compare the obtained result with the proposed method and using the modified electrode, the spiked tap water sample was also analyzed by ICP-MS (24.06 µg L^−1^ (SD: 0.34 µg L^−1^)). These results prove the suitability of G-AgNPs-SPCNFEs for U(VI) determination.

## 4. Conclusions

The proposed green silver and gold nanoparticle-modified screen-printed electrodes were found to be suitable electrochemical devices to determine lead and cadmium concentrations using differential pulse anodic stripping voltammetry. The results showed that the G-AgNPs- and G-AuNPs-modified electrodes were able to perform detection with similar analytical parameters, including the detection and quantification limit, linear range, and sensitivities for Pb(II) determination. Regarding the voltammetric determination of Cd(II), higher detection limits were obtained, and the linear ranges were less restrictive compared to the ones obtained for the Pb(II) determination.

In addition, the G-AgNPs-SPCNFE electrode was successfully used to determine U(VI) as uranyl ion in aqueous samples, and few works with other techniques and worse performances have been published.

In this sense, DPASV was selected, along with G-AgNPs-SPCNFE, and verified in spiked tap water samples. The results are comparable to those obtained by ICP-MS and have good reproducibility.

## Figures and Tables

**Table 1:** Electroactive area (A_T_) calculated using the Randles-Sevcik equation, and charge transfer resistance (R_ct_) values obtained by fitting the Nyquist diagram data for every studied electrode to a Randles circuit.

Electrode	A_T_ (cm^2^)	R_ct_ (Ω)
Bare-SPCNFE	0.00026	638
G-AgNPs-SPCNFE	0.00027	552
G-AuNPs-SPCNFE	0.00028	459

## Data Availability

Not applicable.

## Supplementary Materials

The following are available online at https://www.mdpi.com/article/10.3390/nano13061055/s1, Figure S1. (A–D) TEM micrographs obtained for green silver nanoparticles (G-AgNPs); Figure S2. (A) Voltammograms obtained for U(VI) determination using the G-AuNPs-SPCNFE, and (B) calibration plot. Experimental conditions: acetic acid/acetate buffer 0.1 mol L^−1^ pH 4.5 using an E_d_ of −1.2 V and a td of 300 s.
