# Supplementary material for: Functionalization of Screen-Printed Electrodes with Grape Stalk Waste Extract-Assisted Synthesized Silver and Gold Nanoparticles: Perspectives of Electrocatalytically Enhanced Determination of Uranyl Ion and Other Heavy Metals Ions
Source: Nanomaterials (Basel). 2023 Mar 15;13(6):1055. doi: 10.3390/nano13061055 (PMC10059588; doi:10.3390/nano13061055)
Supplement: Supplementary file 1 [file nanomaterials-13-01055-s001.zip › nanomaterials-2249285-Supplementary Materials.pdf]

Supplementary Materials

Functionalization of Screen-Printed Electrodes with Grape Stalk Waste Extract-Assisted Synthesized Silver and Gold Nanoparticles: Perspectives of Electrocatalytically Enhanced Determination of Uranyl Ion and Other Heavy Metals Ions

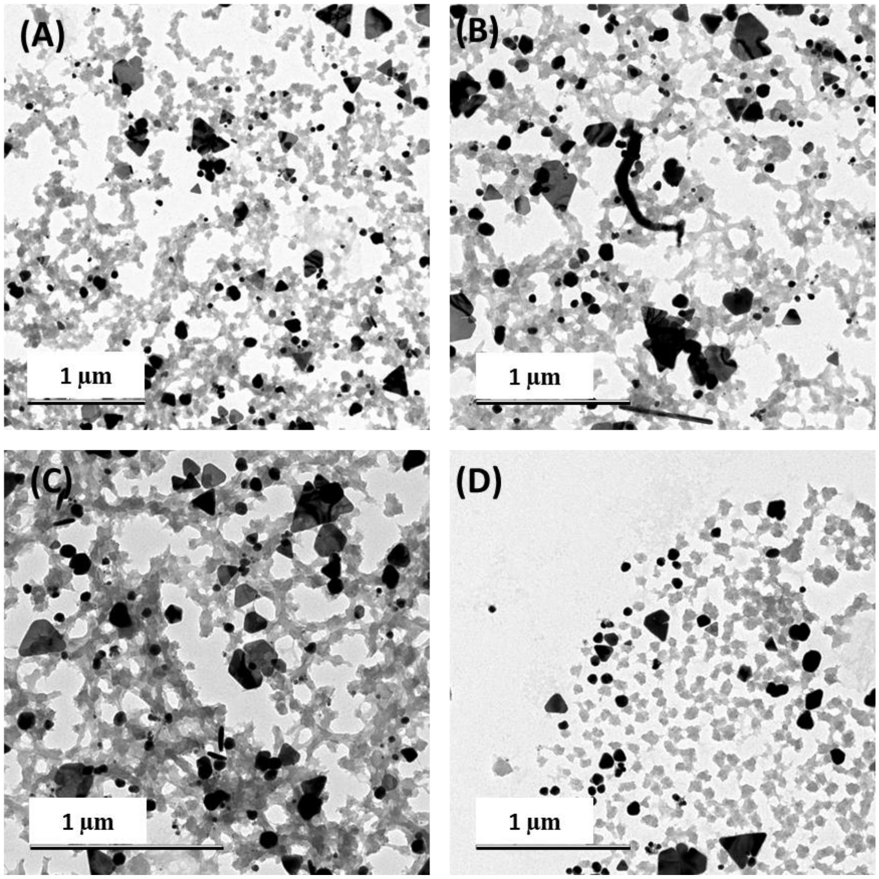

Figure S1. (A–D) TEM micrographs obtained for green silver nanoparticles (G-AgNPs).

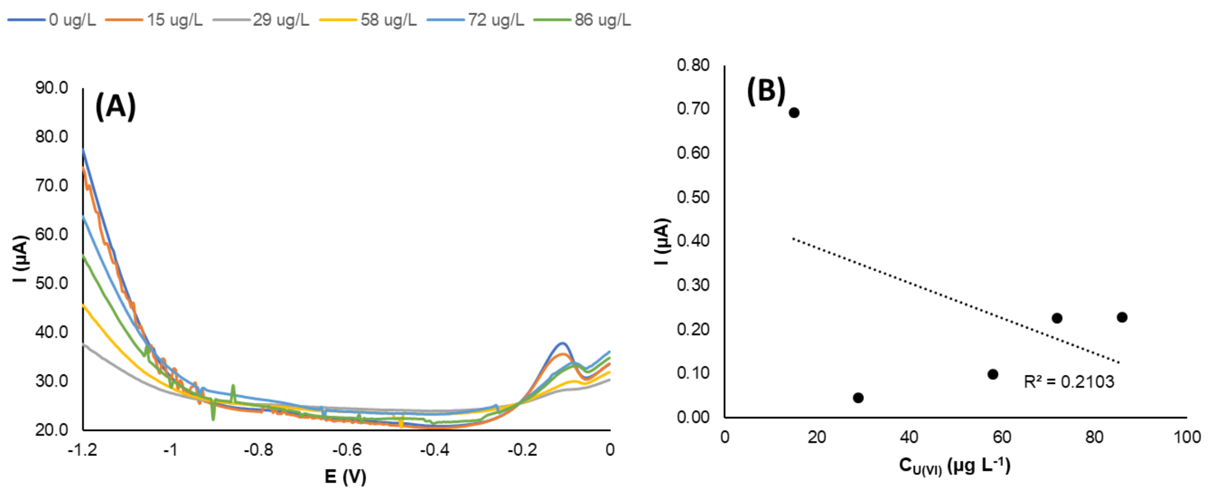

Figure S2. (A) Voltammograms obtained for U(VI) determination using the G-AuNPs-SPCNFE, and (B) calibration plot. Experimental conditions: acetic acid/acetate buffer 0.1 mol L^{-1} pH 4.5 using an Ed of -1.2 V and a td of 300 s.
